# Human Milk Oligosaccharides in Sports Nutrition: Biological Rationale and Current Evidence

**DOI:** 10.3390/nu18203367

**Published:** 2026-10-14

**Authors:** Ke Hu, Kaimeng Zhang, Chenxi Huang, Zhaoguan Wang, Xuanxuan Huang, Yin Li, Lianghui Cheng, Yuxiang Wu

**Affiliations:** 1 Department of Health and Physical Education, Institute of Intelligent Sport and Proactive Health, Jianghan University, Wuhan 430056, China; 251213014002@stu.jhun.edu.cn (K.H.); kaimeng.zhang@outlook.com (K.Z.); 13797634537@163.com (X.H.); liyin@jhun.edu.cn (Y.L.); 2 Synaura Biotechnology (Shanghai) Co., Ltd., Shanghai 201210, China; huangchenxi@synaura.cn; 3 School of Optoelectronic Materials and Technology, Jianghan University, Wuhan 430056, China; wzgname@outlook.com

**Keywords:** sports nutrition, human milk oligosaccharides, exercise, recovery

## Abstract

Established sports-nutrition strategies support fueling, hydration, and muscle protein turnover, but strenuous exercise also challenges gastrointestinal, microbial, immune, metabolic, and musculoskeletal homeostasis. Human milk oligosaccharides (HMOs) are structurally diverse bioactive glycans that can selectively influence gut microbial composition, microbial metabolism, intestinal barrier integrity, and immune function. These properties provide a biological rationale for investigating HMOs in exercise and sports nutrition. This review critically evaluates direct and supportive evidence linking the biological functions of HMOs to exercise physiology. Human evidence is limited to two small trials. One examined supplementation alongside dietary restriction and increased physical activity, limiting conclusions about exercise adaptation; the other found no additional gains in muscle mass, strength, or anaerobic performance during resistance training. Alongside the emerging exercise-related findings, the established effects of HMOs on the gut microbiota and intestinal barrier also suggest a potential role in reducing exercise-associated gastrointestinal disturbances. However, benefits for these disturbances or post-exercise recovery have not been established. HMOs remain candidates for complementing established sports-nutrition strategies, but current evidence is insufficient to recommend supplementation for athletes. Future studies should determine how HMO structure, dose, formulation, and host characteristics influence gastrointestinal resilience, musculoskeletal adaptation, and functional outcomes in physically active populations. Establishing these relationships will be essential for translating HMOs into evidence-based sports-nutrition applications.

## 1. Introduction

Sports nutrition supports not only immediate exercise performance but also the ability to sustain training, adapt to repeated workloads, recover between sessions, and maintain long-term health [1,2,3]. Adequate energy, carbohydrate, and protein intake supports exercise demands, glycogen restoration, and muscle maintenance [4,5], while individualized fluid and electrolyte replacement helps maintain hydration [6,7]. Beyond these fundamental strategies, selected supplements, including creatine, caffeine, dietary nitrate, and specific buffering agents, have relatively strong supporting evidence and may provide additional benefits [8,9]. The broader physiological demands of strenuous exercise have also increased interest in functional ingredients that may complement these approaches by supporting gastrointestinal, metabolic, and immune health [10,11,12].

Strenuous exercise places demands on physiological systems beyond energy metabolism and skeletal muscle function [13,14,15,16], with the gastrointestinal tract being particularly vulnerable during prolonged endurance exercise, repeated high-intensity training, and exercise in the heat [17,18]. Under these conditions, gastrointestinal stress may cause symptoms and epithelial injury, increase intestinal permeability, and alter microbial composition and metabolism [19,20]. These disturbances may compromise carbohydrate and fluid intake and absorption [21,22], while barrier disruption may increase systemic exposure to microbial products and promote inflammatory activation [23,24]. These gastrointestinal and immune disturbances occur alongside the increased requirements for muscle repair and metabolic restoration imposed by repeated training. When recovery or energy availability is inadequate, musculoskeletal adaptation and bone health may also be compromised [3,25]. Together, these interacting demands provide a rationale for nutritional strategies that complement conventional fueling and recovery practices, particularly during intensive training and competition.

Human milk oligosaccharides (HMOs) are a group of bioactive glycans with the potential to contribute to such complementary nutritional strategies. Naturally present in human milk, they comprise more than 200 reported structures, including neutral core, fucosylated, and sialylated oligosaccharides with distinct monosaccharide compositions and glycosidic linkages [22,26]. Their biological importance was first established in early-life nutrition, particularly through their contribution to the development of the intestinal microbiota and mucosal environment [27]. Selected HMOs support microbial growth and metabolism [28] and may influence epithelial barrier function and immune signaling [29,30]. Research has subsequently expanded into adult gastrointestinal and metabolic health [31,32]. The biological processes influenced by HMOs overlap substantially with the gastrointestinal, microbial, metabolic, and immune systems affected by strenuous exercise [33,34]. Emerging experimental and human studies further suggest that selected HMOs may influence exercise capacity, energy metabolism, skeletal muscle function, and musculoskeletal health [35,36,37]. Alongside these biological advances, developments in microbial fermentation, glycosyltransferase engineering, and downstream purification have enabled the scalable production of structurally defined HMOs, making their investigation and application beyond infant nutrition increasingly feasible [38,39,40]. This convergence of biological evidence and production advances provides a timely basis for evaluating HMOs as potential functional ingredients in sports nutrition.

Against this background, this review evaluates the biological rationale, current evidence, and translational potential of HMOs in sports nutrition. The structural characteristics and principal biological functions of HMOs are considered in relation to exercise-associated physiological disturbances. On this basis, direct and supportive evidence concerning exercise capacity, training adaptation, muscle function, skeletal health, and recovery is critically assessed. The review also considers ingredient selection, supplementation strategies, scalable production, safety, regulation, product quality, and priorities for future human studies. By linking mechanistic understanding with exercise-related evidence and practical feasibility, this review provides a framework for determining where HMOs may complement established sports-nutrition strategies and what evidence is still required for their responsible development.

## 2. Methods

This article is a narrative review. The English-language literature was identified through PubMed and Web of Science Core Collection, with the last search conducted on 31 August 2026. Searches covered HMOs and individual HMO structures in relation to exercise, recovery, gut microbiota, intestinal barrier function, immunity, metabolism, muscle, and bone, as well as production, formulation, and safety. Relevant publications were selected by Ke Hu and Lianghui Cheng through assessment of titles, abstracts, and full texts, supplemented by screening the reference lists of relevant publications. The review included original human, animal, and cellular studies, alongside relevant reviews, consensus statements, and regulatory assessments. Both exercise studies and supportive research in developmental, nutritional, aging, and disease models were considered. Duplicate records and publications outside the review scope were excluded. To support representative coverage, original studies were prioritized when evaluating biological effects and efficacy, and positive, null, and inconsistent findings were considered across different HMO structures and experimental settings. Evidence was synthesized narratively, with exercise-related findings distinguished from evidence obtained outside exercise settings.

## 3. Exercise-Associated Physiological Challenges Relevant to HMOs

### 3.1. Gastrointestinal Barrier Dysfunction During Strenuous Exercise

Strenuous exercise can transiently disrupt gastrointestinal integrity and function, particularly when it is prolonged, performed at high intensity, or accompanied by substantial thermal strain. These responses are commonly described as exercise-induced gastrointestinal syndrome, in which circulatory and neuroendocrine pathways interact to impair gastrointestinal function [41]. During exercise, sympathetic activation redistributes blood flow away from the splanchnic circulation toward working skeletal muscle and, especially under heat stress, the skin. The resulting intestinal hypoperfusion can produce epithelial hypoxia and energy depletion, thereby increasing the susceptibility of enterocytes and intercellular junctions to injury [42]. Hyperthermia, dehydration, prolonged exercise duration, and the repetitive mechanical impact associated with running can further aggravate gastrointestinal disturbance, although their relative contributions vary among exercise settings and individuals [43,44].

Evidence of this disruption has been obtained using both circulating biomarkers and permeability tests. A single bout of strenuous exercise can increase intestinal fatty acid-binding protein (I-FABP), a marker of mature enterocyte injury, and alter intestinal permeability measured using orally administered sugar probes [45,46]. Experimental studies further implicate oxidative and nitrosative stress, epithelial apoptosis, and changes in the localization or expression of tight-junction proteins in exercise- and heat-related barrier damage [45,46,47]. However, the magnitude of these responses depends on exercise modality and duration, thermal strain, nutritional intake, training status, and the timing and method of assessment [43].

When barrier integrity is sufficiently compromised, luminal microbial products such as lipopolysaccharide (LPS) may enter the circulation. Recognition of LPS by LPS-binding protein, cluster of differentiation 14 (CD14), and Toll-like receptor 4 (TLR4) can amplify innate immune and systemic inflammatory signaling [48]. Circulating endotoxin and associated inflammatory responses have been reported, particularly after ultra-endurance exercise and exertional heat stress [49]. Microbial-product translocation may therefore provide a context-dependent link between intestinal barrier damage and systemic inflammation rather than representing an inevitable response to strenuous exercise.

Gastrointestinal symptoms, including nausea, abdominal pain, bloating, reflux, urgency, and diarrhea, are also common during endurance events and may restrict carbohydrate and fluid intake [34,41,50]. These symptoms arise from interacting circulatory, neuroendocrine, mechanical, and nutritional factors and do not consistently correspond to the extent of epithelial injury or increased permeability [33,51]. Symptom assessment and biological markers should therefore be treated as complementary outcomes. Both symptomatic and subclinical gastrointestinal disturbances may reduce tolerance to prolonged exercise and increase exposure to luminal microbial products. Because the intestinal environment is also shaped by microbial composition and metabolic activity, the relationship between exercise and the gut microbiota warrants further consideration.

### 3.2. Exercise, Gut Microbiota, and Microbial Metabolism

The relationship between exercise and the gut microbiota is potentially bidirectional: physical activity can modify microbial composition and function, whereas microbial metabolites may, in turn, influence host metabolism and exercise-related phenotypes. Regular exercise training has been associated with greater microbial diversity, enrichment of short-chain fatty acid (SCFA)-producing taxa, and altered microbial metabolic capacity [52,53]. However, these responses vary substantially according to exercise modality and intensity, host metabolic status, habitual diet, and baseline microbiota composition [54,55,56]. In a controlled intervention involving previously sedentary adults, six weeks of endurance exercise altered microbial composition and increased fecal SCFA concentrations, particularly in lean participants; these changes were partially reversed after participants returned to a sedentary lifestyle [57]. The increase in SCFAs suggests a potentially beneficial change in microbial metabolism, although these findings alone do not establish improved intestinal health or exercise performance. Cross-sectional differences between athletes and sedentary individuals should nevertheless be interpreted cautiously because diet, energy intake, body composition, medication use, and other lifestyle factors may contribute substantially to the observed microbial profiles [53].

Microbial metabolites also provide a functional link between exercise, intestinal physiology, and systemic metabolism. SCFAs, including acetate, propionate, and butyrate, influence host physiology through both metabolic and signaling pathways. Butyrate provides energy for colonocytes, while SCFAs also activate G-protein-coupled receptors to regulate intestinal, immune, and metabolic functions [58]. In addition, butyrate and propionate can inhibit histone deacetylases, altering histone acetylation and gene expression to influence immune-cell differentiation, including regulatory T-cell development [58,59]. Through these metabolic, receptor-mediated, and epigenetic mechanisms, SCFAs contribute to epithelial energy metabolism, intestinal barrier maintenance, immune regulation, glucose and lipid metabolism, and whole-body energy homeostasis [60,61,62]. Changes in microbial metabolite production could therefore influence exercise performance by modifying energy availability and metabolic signaling, while supporting intestinal function and limiting excessive inflammatory responses.

Higher cardiorespiratory fitness has been associated with increased microbial diversity, greater abundance of butyrate-producing taxa, and higher fecal butyrate concentrations [63]. Scheiman et al. observed an increase in Veillonella abundance after marathon running and demonstrated that *Veillonella atypica* (*V. atypica*) can use lactate as a carbon source and convert it through the methylmalonyl-CoA pathway into propionate [64]. Colonization with *V. atypica*, or intrarectal administration of propionate, prolonged treadmill running time in mice, providing experimental evidence that microbial metabolism can influence exercise capacity [64]. More recently, microbiota-derived fatty acid amides were shown to regulate exercise motivation and performance through a gut–brain sensory pathway in mice [65]. These studies support the possibility of microbial metabolites may influence exercise capacity through metabolic and neural pathways, but whether these mechanisms improve performance or recovery in humans remains uncertain.

### 3.3. Immune and Inflammatory Perturbations

Strenuous exercise induces a coordinated but generally transient immune and inflammatory response. Its magnitude and duration depend on exercise intensity and duration, training status, energy availability, environmental conditions, and the timing of sample collection [66,67]. Acute exercise rapidly mobilizes leukocyte populations into the circulation, followed by their redistribution to peripheral tissues during recovery [13,68]. The post-exercise decline in circulating lymphocytes and natural killer cells is now considered to reflect immune-cell trafficking rather than generalized immune suppression [66]. Accordingly, the term exercise-associated immune perturbation is more appropriate than exercise-induced immunosuppression.

Cytokine responses form an important component of this process. Interleukin-6 (IL-6) released from contracting skeletal muscle increases markedly during prolonged exercise and contributes to substrate mobilization and inter-organ metabolic communication [69]. It can also promote counter-regulatory mediators, including interleukin-10 (IL-10) and interleukin-1 (IL-1) receptor antagonist, thereby helping to control and resolve the acute inflammatory response [70]. By contrast, increases in classical pro-inflammatory mediators such as Tumor Necrosis Factor-alpha (TNF-α) are less consistent and may be more evident when strenuous exercise is accompanied by substantial tissue damage, intestinal endotoxin translocation, heat stress, or inadequate recovery [13,16,71]. Exercise-induced inflammation should therefore not be regarded as uniformly detrimental, because appropriately regulated inflammatory signaling is necessary for tissue repair and training adaptation.

Problems may arise when repeated high training loads are combined with insufficient recovery, inadequate energy intake, sleep disruption, or other physiological and psychological stressors. Under these conditions, immune and inflammatory disturbances may persist beyond the normal recovery period and contribute to illness symptoms, delayed recovery, and reduced training availability [15,72]. Because intestinal barrier disruption and microbial product translocation can amplify inflammatory signaling, the gut may represent one—but not the only—link between exercise stress and systemic immune homeostasis [41]. This provides a rationale for investigating nutritional interventions that influence intestinal and immune regulation; however, whether HMOs can meaningfully attenuate exercise-associated immune perturbations remains to be established.

### 3.4. Metabolic and Recovery Demands

Recovery from strenuous exercise requires the coordinated restoration of fuel stores, fluid and electrolyte balance, muscle repair, and redox, inflammatory, gastrointestinal, and immune homeostasis. The contribution of each process varies with the exercise performed, environmental conditions, training status, and the time available before the next session [10]. In this review, recovery refers to the restoration of physiological function and exercise capacity after an exercise bout. Training adaptation refers to sustained changes resulting from repeated training over weeks or months. Resilience describes the capacity to maintain function and continue training despite repeated exercise stress. Although related, these concepts require different outcomes, and changes in isolated biomarkers do not by themselves establish improvements in any of them.

Carbohydrate, fluid, and protein intake remain the foundation of established recovery nutrition. Carbohydrate ingestion during prolonged exercise helps maintain exogenous carbohydrate availability and blood glucose, whereas post-exercise intake supports glycogen resynthesis, particularly when the recovery interval is short [3,73,74]. Fluid and electrolyte replacement is used to restore exercise-induced losses, although requirements vary substantially with sweat rate, environmental conditions, exercise duration, and individual tolerance [75]. Adequate dietary protein and essential amino acids stimulate muscle protein synthesis and support training adaptation, but evidence that additional protein supplementation consistently accelerates the recovery of muscle function, soreness, and indirect markers of muscle damage is less uniform [76,77].

Beyond these foundational nutritional practices, several adjunctive supplements have been investigated for specific aspects of performance and recovery. Isolated branched-chain amino acid supplementation may reduce perceived muscle soreness in some settings, although its effects on muscle function, endurance performance, and broader recovery outcomes remain inconsistent [78,79]. Creatine monohydrate is strongly supported for increasing intramuscular creatine availability and improving repeated high-intensity exercise capacity and resistance-training adaptation [80,81]. However, its effects on recovery from exercise-induced muscle damage are less consistent, with systematic reviews reporting changes in selected biochemical markers but little reproducible improvement in soreness, range of motion, or muscle function [82]. Polyphenol-rich interventions, particularly tart cherry products, may improve selected measures of soreness, muscle function, oxidative stress, and inflammation after strenuous exercise, although outcomes vary with the exercise model, supplementation protocol, training status, and recovery measure [83,84]. A narrative review of athletes discussed their potential to modulate exercise-induced oxidative stress and antioxidant status, while emphasizing that the evidence does not support uniform supplementation recommendations [85]. A systematic review in team-sport athletes likewise reported possible benefits for muscle function and soreness during recovery, although the certainty of evidence ranged from moderate to very low [86]. These supplements should therefore be selected according to the specific performance or recovery outcome for which they have been evaluated rather than regarded as universally effective recovery strategies.

These differences in function should not be interpreted as shortcomings of established recovery strategies. Carbohydrate, fluid, protein, creatine, and polyphenol interventions address distinct and practically important aspects of fueling, hydration, muscle metabolism, and recovery. However, they were not primarily developed to regulate the interconnected gastrointestinal, microbial, and immune disturbances that may accompany strenuous exercise. This has increased interest in complementary nutritional approaches that act at the gut–microbiota–immune interface. Probiotics are among the most widely studied examples and may provide strain- and dose-dependent benefits for selected gastrointestinal, respiratory, and immune outcomes in athletes [87]. Their effects on microbial composition, inflammatory markers, recovery, and exercise performance nevertheless remain variable. A recent meta-analysis found that probiotic supplementation increased post-exercise IL-10 but did not consistently alter interleukin-1β (IL-1β), IL-6, interleukin-8 (IL-8), TNF-α, or interferon-γ (IFN-γ), indicating that systemic responses to gut-directed interventions are context-dependent [71].

HMOs are relevant to this interface because their biological activities extend beyond microbiota modulation. Most HMOs resist digestion in the upper gastrointestinal tract and reach the colon, where selected bacteria, particularly HMO-utilizing *Bifidobacterium* species, can use them as growth substrate [71,88,89]. Their utilization may support microbial cross-feeding and alter metabolite production [90]. Selected HMOs may also influence intestinal barrier function and epithelial or immune signaling through microbiota-dependent and direct pathways [91,92]. This combination of microbial, barrier, and immune effects provides a broader rationale for investigating HMOs as complements to established recovery nutrition. Their effects are likely to depend on HMO structure, dose, host characteristics, and baseline microbiota, while direct evidence under exercise conditions remains limited. The following section describes the structural characteristics and biological functions underlying this potential relevance.

## 4. HMO Structure and Biological Actions Relevant to Exercise Physiology

HMOs are a diverse group of bioactive glycans. They are best known for their functions in early-life nutrition. However, their effects on the gut microbiota, intestinal barrier, and immune system may also be relevant to adults. These biological targets overlap with several systems that can be disturbed by strenuous exercise. This section describes the structure and gastrointestinal fate of HMOs, followed by their effects on the gut–microbiota–immune interface.

### 4.1. Structural Diversity and Gastrointestinal Fate

HMOs are among the most abundant solid components of human milk after lactose and lipids [93,94]. Their total concentration is generally higher in colostrum than in mature milk. Concentrations of approximately 20–25 g/L have been reported in colostrum, whereas mature milk commonly contains around 5–20 g/L [92,93,95]. However, values differ considerably among individuals and across the stages of lactation [95]. More than 200 HMO structures have been identified. Milk from other mammals also contains oligosaccharides, but human milk has an unusually high concentration and structural diversity [26,96].

**Figure 1:**
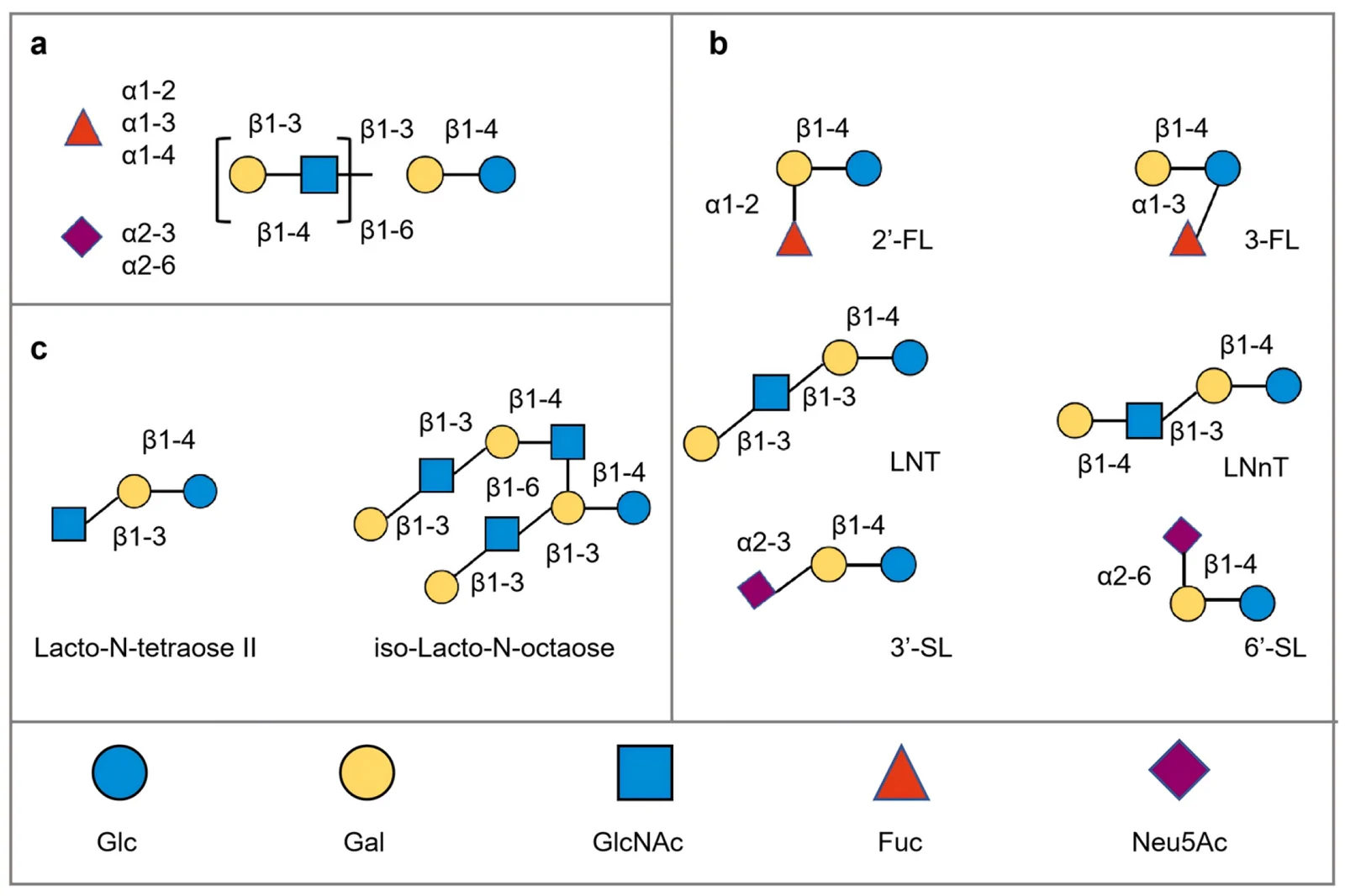
Structural framework and classification of HMOs. (**a**) Common monosaccharide building blocks and core structural features of HMOs; (**b**) Representative linear and branched HMO architectures; (**c**) Major HMO classes based on fucosylation and sialylation, including neutral non-fucosylated, neutral fucosylated, and acidic sialylated structures. Abbreviations: Glc, glucose; Gal, galactose; GlcNAc, N-acetylglucosamine; Fuc, fucose; Neu5Ac, N-acetylneuraminic acid; 2′-FL, 2′-fucosyllactose; 3-FL, 3-fucosyllactose; LNT, lacto-N-tetraose; LNnT, lacto-N-neotetraose; 3′-SL, 3′-sialyllactose; 6′-SL, 6′-sialyllactose.

HMOs are built from five basic monosaccharide units: ᴅ-glucose (Glc), ᴅ-galactose (Gal), N-acetylglucosamine (GlcNAc), ʟ-fucose (Fuc), and sialic acid (N-acetylneuraminic acid, NeuAc) [97,98,99]. All HMOs contain lactose at their reducing end; building blocks are connected through various glycosidic linkages and branching patterns, generating a highly diverse structural network (Figure 1a). Based on structural features, HMOs can be broadly categorized as linear and branched (Figure 1b), and can be further classified into neutral fucosylated type [100], neutral non-fucosylated type [101], and acidic sialylated type [102]. Representative structures include 2′-fucosyllactose (2′-FL), 3-fucosyllactose (3-FL), lacto-N-tetraose (LNT), lacto-N-neotetraose (LNnT), 3′-sialyllactose (3′-SL), and 6′-sialyllactose (6′-SL). These structures constitute a major proportion of total HMOs in human milk (Figure 1c).

The HMO profile is not fixed; it is influenced by the mother’s secretor and Lewis blood-group status, stage of lactation, geographical background, and other biological factors [103]. For example, the presence and concentration of 2′-FL are strongly affected by the activity of fucosyltransferase2 [104]. Despite these differences in composition, most HMOs share a similar gastrointestinal fate. They resist digestion by human enzymes in the upper gastrointestinal tract and reach the lower intestine largely intact, where they become available for utilization by selected gut bacteria [105]. A smaller fraction may be absorbed and detected in the circulation or urine, although the physiological importance of this pathway remains unclear [106,107].

Most research on HMOs has focused on infants, but purified HMOs have also been studied in adults. In a randomized controlled trial in healthy adults, supplementation with chemically produced 2′-FL, LNnT, or their combination for two weeks was well tolerated and changed the gut microbiota [31]. A later study using a more complex HMO concentrate from pooled donor human milk also reported dose-dependent changes in the adult gut microbiota [92]. These findings show that the adult gut microbiota can also respond to oral HMO supplementation, and microbial utilization is likely to be one of the main pathways through which they act in adults.

### 4.2. Effects on the Gut Microbiota and Microbial Metabolism

HMOs do not support the growth of all gut bacteria equally. Marcobal et al. compared the ability of several gut-associated bacteria to use natural HMO mixtures isolated from pooled donor human milk HMOs as a carbon source. *Bifidobacterium longum* subsp. *infantis* (*B. infantis*) and several *Bacteroides* strains consumed HMOs efficiently, whereas the tested strains of *Enterococcus*, *Streptococcus*, *Veillonella*, *Clostridium*, and *Escherichia coli* showed little or no growth [108]. These findings indicate that HMOs act as selective microbial substrates rather than general carbohydrates for the whole gut community.

Although bifidobacteria can efficiently consume HMOs, they utilize these compounds through different pathways. *B. longum* subsp. *infantis* carries several transporters and intracellular glycosidases that allow it to import and degrade a broad range of HMO structures [109]. In contrast, *Bifidobacterium bifidum* (*B. bifidum*) mainly uses extracellular enzymes to break HMOs into smaller sugars before uptake [28]. Other species have a more restricted capacity. For example, James et al. showed that *Bifidobacterium breve* (*B. breve*) UCC2003 could metabolize both LNT and LNnT, but used partly different transport and enzymatic pathways for the two structures [110]. More recently, Sanchez-Gallardo et al. tested 23 strains of *Bifidobacterium pseudocatenulatum* (*B. pseudocatenulatum*) and found marked differences in their ability to grow on 2′-FL, 3-FL, LNT, and LNnT. Genes required for LNT degradation were widely distributed, whereas genes required for fucosylated HMO utilization were present in only a subset of strains [111]. Thus, HMO utilization depends on both the HMO structure and the genetic capacity of the bacterial strain.

HMO degradation can also support bacteria that cannot use intact HMOs. Lawson et al. examined 19 bifidobacterial strains isolated from breastfed infants [112]. Nine strains used 2′-FL and 12 used LNnT, but this ability varied even among strains isolated from the same infant. HMO-utilizing strains released fucose, galactose, N-acetylglucosamine, and acetate into the culture medium. Conditioned-medium and co-culture experiments showed that some non-HMO-utilizing bifidobacteria could grow on these released products [112]. This type of cross-feeding allows several bacterial strains to share the products of HMO degradation. Cross-feeding also changes the metabolites produced by the wider microbial community. Schwab et al. demonstrated that during 2′-FL and 3-FL degradation, *B. longum* subsp. *infantis* produced acetate, lactate, and 1,2-propanediol; *Eubacterium hallii* (*E. hallii*) then used these products and generated butyrate, propionate, and formate [113]. Cheng et al. observed that while *Bifidobacterium* strains efficiently utilized various HMOs in monoculture to produce acetate and lactate, their growth was significantly enhanced in co-culture with *Faecalibacterium prausnitzii* (*F. prausnitzii*), a syntrophic interaction where *F. prausnitzii* likely consumed lactate to generate butyrate [114]. Therefore, HMO fermentation metabolites may be produced indirectly through microbial cross-feeding rather than directly by the primary HMO-utilizing bifidobacteria.

The microbial effects of HMOs may also depend on other dietary components present during fermentation. Potential complementary interactions with polyphenols have been proposed through changes in HMO-utilizing bacteria and microbial cross-feeding, which may influence SCFA production [115]. Direct fermentation studies provide additional evidence for interactions with other nondigestible carbohydrates. Combining GOS with 2′-FL altered substrate utilization and fermentation kinetics, although it did not ensure complete 2′-FL degradation [116]. Combinations of 2′-FL, oligofructose, and β-glucan also increased propionate and butyrate production compared with individual substrates in vitro [117]. These findings indicate that the dietary context can shape HMO-associated microbial metabolism, but combined effects depend on the ingredients and microbial community and should not automatically be interpreted as synergy.

Evidence from human studies shows that HMOs can also modify the adult gut microbiota. In a randomized controlled trial involving 100 healthy adults, Elison et al. administered 2′-FL, LNnT, or a 2′-FL/LNnT mixture at doses of 5–20 g/day for two weeks and observed an increase in the relative abundance of *Bifidobacterium* [31]. Jacobs et al. subsequently provided a complex HMO concentrate at 1.8–18 g/day to 32 healthy adults for seven days. The intervention caused a dose-dependent expansion of *Bifidobacterium* and changes in microbial gene content. The microbial community continued to change after supplementation ended, indicating that the response involved ecological succession rather than only short-term substrate use [92]. Similar changes have been observed in adults with gastrointestinal disorders. In a study of 58 patients with irritable bowel syndrome, four weeks of supplementation with a 4:1 mixture of 2′-FL and LNnT increased both fecal and mucosal bifidobacteria, particularly *Bifidobacterium adolescentis* (*B. adolescentis*) and *Bifidobacterium longum* (*B. longum*) [118]. These findings indicate that HMO supplementation can increase the abundance of adult-associated bifidobacteria.

Adult microbial responses to HMOs are likely to depend on baseline microbiota composition, HMO structure, dose, intervention duration, background diet, and microbial cross-feeding. Current studies show that HMOs can alter microbial composition and metabolism in adults, but whether these changes produce meaningful benefits under exercise-related stress remains unclear. HMO-induced microbial changes and metabolites may also influence the intestinal epithelium, providing a mechanistic link to barrier function and host–microbe interactions.

### 4.3. Intestinal Barrier Function and Host–Microbe Interactions

The microbial changes may also influence the intestinal barrier; HMOs can affect the barrier function indirectly through microbial fermentation or directly through interactions with intestinal epithelial cells. Šuligoj et al. proved that fermentation of 2′-FL and LNnT by adult gut microbiota increased bifidobacteria and altered SCFA production, yielding metabolites that reduced epithelial permeability, decreased IL-6 secretion, and upregulated tight junction proteins claudin-5 (CLDN5) and claudin-8 (CLDN8) in Caco-2 and human colon organoid models [27]. These results support a role for microbial metabolites in barrier regulation. Paone et al. found that 2′-FL supplementation changed mucus production, mucin glycosylation, and the microbial capacity to degrade mucin glycans. These effects occurred together with changes in the gut microbiota and reduced weight gain and glucose intolerance in mice fed a high-fat diet [119]. Natividad et al. evaluated six individual HMOs and their multi-component blends in cytokine-challenged Caco-2/HT29-MTX monolayers. They found that HMO blends dose-dependently mitigated TNF-α/IFN-γ-induced barrier dysfunction, with 2′-FL demonstrating the strongest protective efficacy [120].

In addition to changing the physical barrier, HMOs can interfere with the first contact between intestinal pathogens and host cells. Many pathogens begin infection by binding to specific glycans on the epithelial surface. Some HMOs contain similar glycan structures and can compete for this binding, which is commonly described as a soluble decoy receptor effect [121]. Ruiz-Palacios et al. showed that *Campylobacter jejuni* (*C. jejuni*) recognizes the fucosylated H(O) antigen on intestinal cells; HMOs containing α1,2-linked fucose inhibited bacterial binding to human intestinal epithelial cells and reduced infection [122]. Weichert et al. later showed 2′-FL and 3-FL reduce the adhesion of *C. jejuni*, enteropathogenic *Escherichia coli*, Salmonella enterica serovar *Fyris*, and *Pseudomonas aeruginosa* [123]. Studies on breastfed infants confirm that these interactions are clinically relevant. Morrow et al. followed 93 mother–infant pairs and measured fucosylated oligosaccharides in maternal milk; higher 2′-FL exposure was specifically associated with fewer episodes of *Campylobacter*-related diarrhea, whereas another fucosylated HMO, lacto-N-difucohexaose I, was associated with a lower incidence of *Calicivirus*-related diarrhea [124].

These anti-adhesive effects represent one part of the broader interaction between HMOs, intestinal microorganisms, and the host. By maintaining epithelial barrier integrity or limiting microbial adhesion, selected HMOs may reduce epithelial exposure to pathogens, toxins, and other microbial products. Some HMOs also interact directly with epithelial or immune cells. The effects of HMOs on the intestinal barrier are therefore closely connected to their regulation of immune and inflammatory responses.

### 4.4. Immune and Inflammatory Regulation

The intestinal barrier and the immune system are closely connected. HMOs may influence immune responses through two routes. Some HMOs act directly on epithelial or immune cells. Others act indirectly by changing the gut microbiota and the metabolites produced by these microorganisms. He et al. showed that 2′-FL reduced the expression of LPS receptor CD14 in human intestinal epithelial cells; it also reduced IL-8 production after stimulation with LPS or enterotoxigenic *Escherichia coli* [125]. In another study, 2′-FL reduced the inflammatory response induced by *Campylobacter jejuni* in human epithelial cells and mouse intestinal tissue [126]. These findings suggest that 2′-FL can reduce epithelial responses to selected bacterial stimuli. Direct epithelial effects also differ among HMO structures. Cheng et al. compared seven individual HMOs in human intestinal epithelial cells exposed to TNF-α. Among the HMOs tested, 3-FL, LNnT, and Lacto-N-difucohexaose (LDFT) reduced TNF-α-induced IL-8 production in fetal intestinal epithelial cells [127]. Importantly, the same effects were not consistently observed in adult T84 epithelial cells. Lacto-N-triose II increased IL-8 production in this cell model, consistent with its reported ability to activate Toll-like receptors (TLRs) [128]. These results show that the response depends on both the HMO structure and the type of epithelial cell being studied.

HMOs may also interact directly with immune cells. In human monocyte-derived dendritic cells, a pooled HMO mixture changed the response to LPS. The HMO mixture increased the production of IL-10, interleukin-27 (IL-27), and IL-6 but not interleukin-12p70 (IL-12p70) and TNF-α, and promoted a more regulatory cluster of differentiation 4^+^ (CD4^+^) T-cell response [129]. Because this study used an HMO mixture, the effects cannot be assigned to a single HMO structure. Studies using individual HMOs provide further evidence of structure-dependent immune responses. Boll et al. compared various HMOs in epithelial and immune-cell models, demonstrating that 3′-SL and 6′-SL elicited the most potent immunomodulatory effects. Specifically, these sialylated HMOs upregulated IL-10, IL-12p70, and interleukin-23 (IL-23) in dendritic cells and macrophages, while enhancing IFN-γ and interleukin-17A (IL-17A) but suppressing interleukin-13 (IL-13) in CD4^+^ T cells [130]. These changes indicate a shift in the immune response rather than a general reduction in inflammation. A recent macrophage study produced a similar conclusion. Jepsen et al. examined the effects of 2′-FL, LNnT, and 6′-SL on human monocyte-derived macrophages exposed to *Staphylococcus aureus*. Of the HMOs tested, 6′-SL produced the strongest response. It increased Nuclear Factor kappa-light-chain-enhancer of activated B cells (NF-κB) activation, bacterial uptake, and the production of TNF-α, IL-6, IL-8, IFN-γ, and IL-1β [131].

In addition to these direct effects, HMOs also regulate immunity indirectly through the gut microbiota. As described above, HMOs modulate bacterial metabolites. These fermentation products, such as short-chain fatty acids, regulate epithelial and immune cells by interacting with G-protein-coupled receptors and inhibiting histone deacetylases. Through these pathways, they can influence epithelial metabolism, cytokine production, macrophage activity, and T-cell differentiation [132]. For example, butyrate promoted the differentiation of colonic regulatory T cells through changes in histone acetylation at the Foxp3 locus in mice [133]. These findings explain a possible route from HMO fermentation to immune regulation. However, they should be interpreted carefully. The amount and pattern of short-chain fatty acids produced from HMOs depend on the HMO structure, the bacterial strains present, and microbial cross-feeding [91,105].

**Figure 2:**
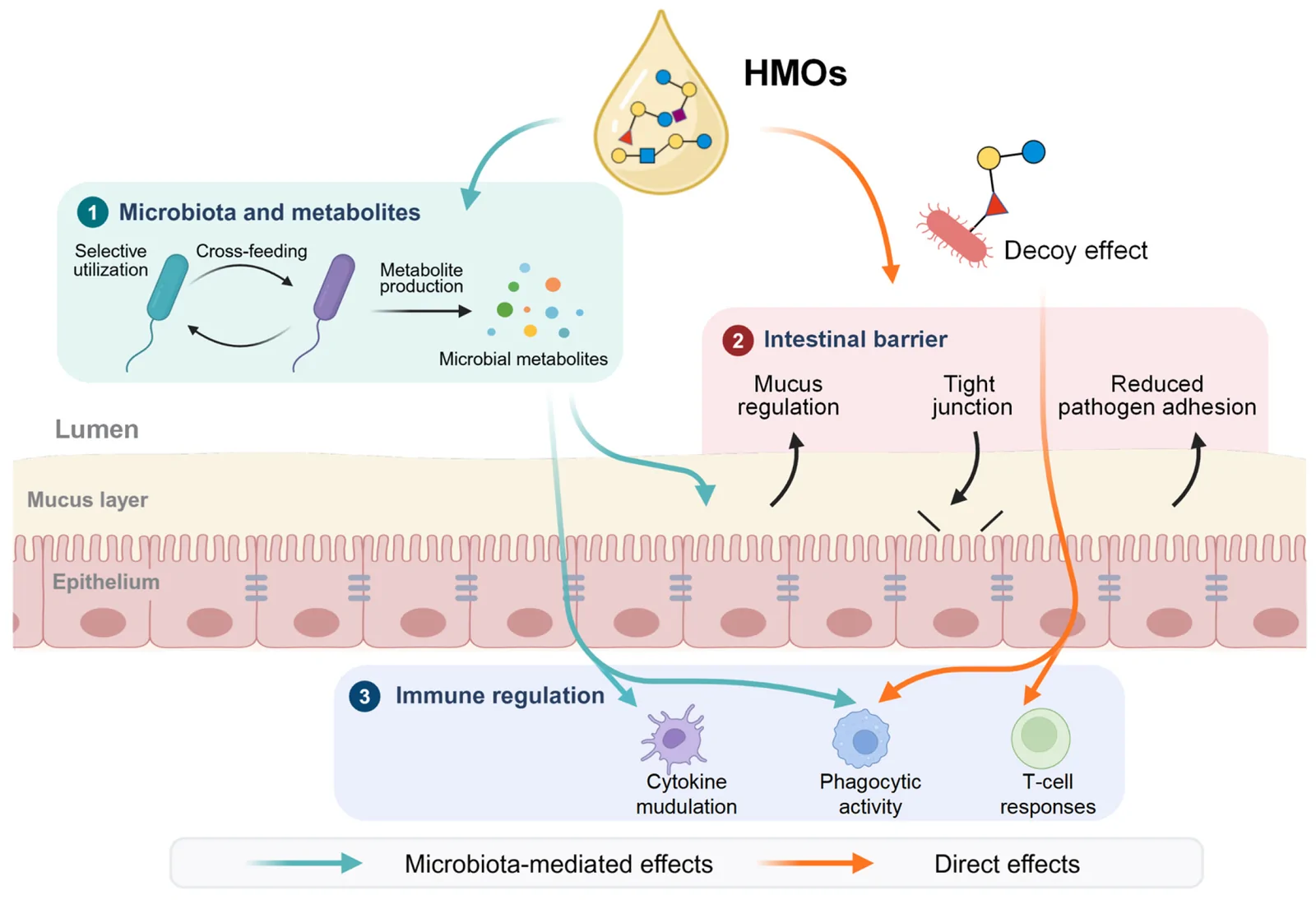
General biological actions of HMOs. HMOs influence gut microbial metabolism, intestinal barrier function, and immune responses through microbiota-mediated effects and direct interactions. HMOs, human milk oligosaccharides.

Adult supplementation studies illustrate this uncertainty. In healthy adults, a complex HMO concentrate produced dose-dependent changes in the gut microbiota. These changes were accompanied by increases in circulating Transforming growth factor-β (TGF-β) after seven days and IL-10 after fourteen days, whereas TNF-α and IFN-γ did not change significantly [92]. More recently, a six-week randomized controlled trial of 2′-FL in healthy older adults increased *Bifidobacterium*, but did not meet its primary endpoint for cytokine responses [32]. These findings show that HMOs can regulate immune responses through several direct and microbiota-mediated pathways. Together with their effects on the gut microbiota and intestinal barrier, these actions provide a biological basis for considering whether HMOs may be useful under conditions that disturb gastrointestinal and immune homeostasis. The direct and microbiota-mediated biological actions of HMOs are summarized in Figure 2.

### 4.5. Functional Relevance to Exercise Physiology

The potential relevance of HMOs to exercise physiology arises from the correspondence between their biological functions and the gastrointestinal and immune disturbances associated with strenuous exercise. Prolonged endurance exercise, repeated high-intensity training, and exercise in the heat can alter microbial metabolism, compromise intestinal barrier integrity, and activate local and systemic inflammatory pathways [134,135]. Because these processes influence one another, HMOs may be more appropriately considered modulators of the gut–microbiota–immune interface than direct sources of energy or conventional ergogenic aids.

Exercise-related changes in the gut microbiota are shaped by exercise intensity, training status, diet, environmental exposure, and sampling time, and therefore vary considerably among individuals [41]. Changes in food intake, carbohydrate availability, gastrointestinal transit, and physiological stress during intensive training or competition may further affect microbial substrate use and metabolite production. Selected HMOs can provide substrates for specific gut bacteria, promote microbial cross-feeding, and alter the production of acetate, lactate, and other metabolites [111,118]. These functions may help preserve microbial metabolic activity under exercise-related stress. Accordingly, the relevance of HMOs should not be judged by whether they shift the microbiota toward a single predefined composition, but by whether they maintain microbial functions that support intestinal homeostasis during exercise-related physiological stress.

The intestinal barrier provides a more direct point of correspondence. During prolonged or heat-stressed exercise, splanchnic hypoperfusion, hyperthermia, and mechanical stress can damage epithelial cells and increase intestinal permeability [16,44]. The resulting passage of luminal microbial products into the circulation may amplify gastrointestinal symptoms and systemic inflammatory signaling. Selected HMOs and their fermentation products have been shown to influence mucus organization, epithelial resistance, tight-junction regulation, and cellular responses to inflammatory stimuli [119,130,136]. These effects provide a biological rationale for investigating whether HMOs can improve intestinal resilience during strenuous exercise.

Strenuous exercise induces coordinated changes in immune-cell trafficking, cytokine production, and inflammatory signaling. Repeated strenuous exercise combined with insufficient recovery may prolong inflammatory activity and temporarily impair some aspects of immune defense [16]. Selected HMOs have been shown to influence cytokine production, macrophage activation and phagocytosis, and T-cell-associated responses [125,127,129]. HMOs may therefore affect both inflammatory control and host defense. Their potential value is not the broad suppression of inflammation, but the prevention of excessive or prolonged immune disturbance while preserving normal immune function. In exercise nutrition, the central question is whether this regulation can promote timely immune recovery between repeated bouts of strenuous exercise.

Together, these mechanisms provide a biological rationale for investigating HMOs as complements to established sports-nutrition strategies. However, most supporting evidence comes from studies outside exercise settings. Direct exercise studies have not consistently assessed gastrointestinal symptoms, epithelial injury, intestinal permeability, microbial-product translocation, or changes in the microbiota and its metabolites alongside functional outcomes. Whether these intestinal effects improve gastrointestinal tolerance or recovery during strenuous exercise therefore remains an untested hypothesis. Changes in exercise capacity or muscle function alone do not establish a gut-mediated mechanism. Findings for one HMO cannot be generalized to other structures or mixtures. The following section evaluates the studies that have directly examined HMOs in relation to exercise capacity, training responses, muscle function, and recovery.

## 5. Evidence Linking HMOs to Exercise, Training, and Recovery

**Figure 3:**
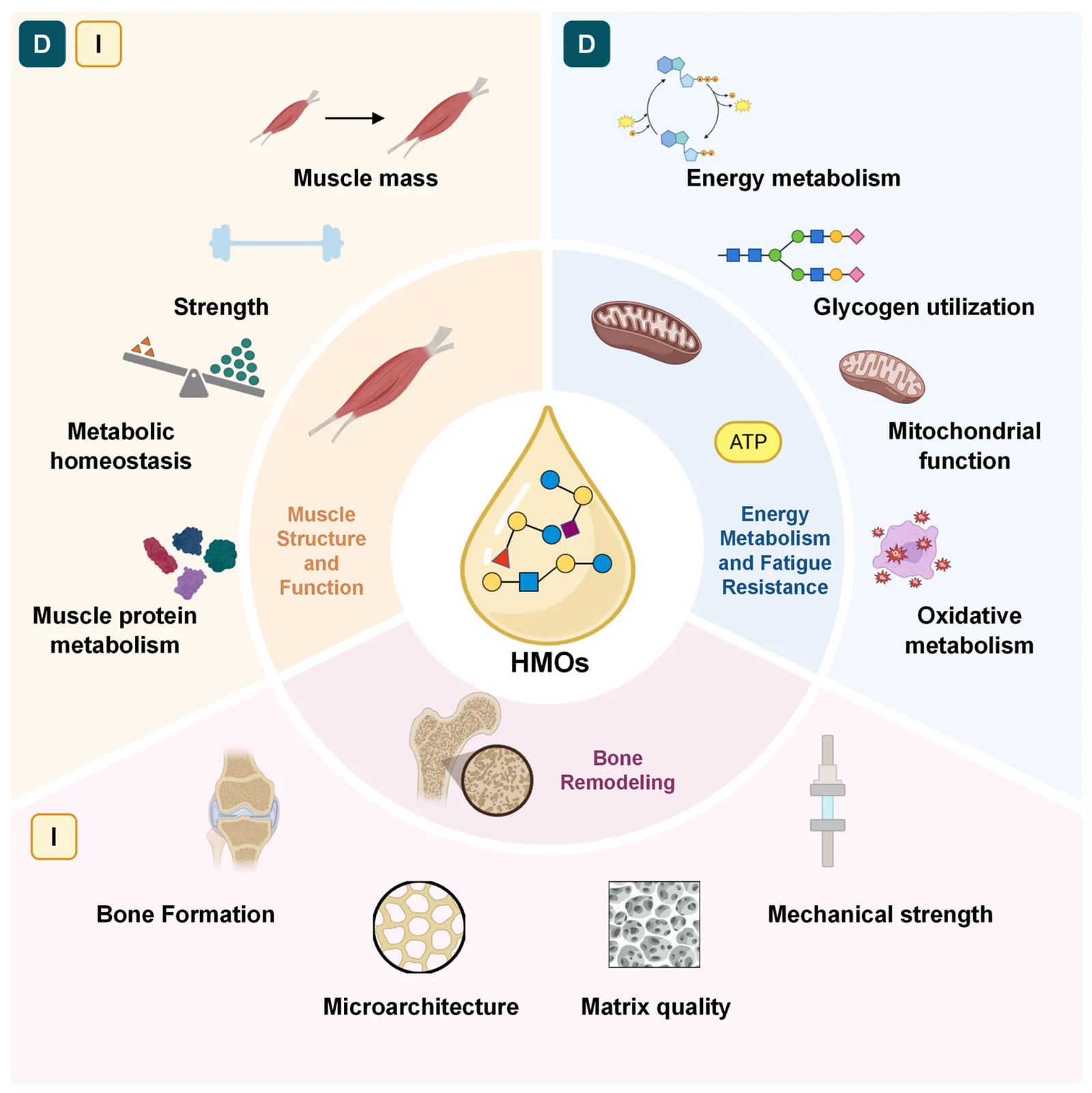
Roles of HMOs in exercise-related metabolism and musculoskeletal function. D: direct exercise-related evidence from experimental models or human interventions; I: indirect evidence from non-exercise settings. Positive muscle and performance findings are predominantly preclinical; benefits in human athletes have not been demonstrated. Abbreviation: ATP, adenosine triphosphate; HMOs, human milk oligosaccharides.

Evidence for the potential role of HMOs in sports nutrition comes from exercise and training studies, research on muscle and bone under disease, aging, or nutritional stress, and broader investigations of microbial and intestinal functions (Table 1). These studies contribute different levels of support. Exercise interventions provide evidence specific to the model or population examined, while findings from other settings help explain how HMOs might influence exercise-related processes. This section evaluates their relevance to exercise capacity, musculoskeletal health, and recovery, while distinguishing observed effects from proposed applications (Figure 3).

### 5.1. Direct Evidence from Exercise and Training Studies

Studies involving exercise or training provide the most direct evidence for the potential relevance of HMOs to sports nutrition, although most have been conducted in experimental models. Their outcomes include endurance, muscle strength, substrate metabolism, muscle-fiber phenotype, and mitochondrial protein expression, with conclusions limited to the model or population examined. Arellano Spadaro et al. investigated 3′-SL and 6′-SL using swimming exercise in the nematode *Caenorhabditis elegans* (*C. elegans*) as a model of endurance-type exercise [137]. In *C. elegans*, both 3′-SL and 6′-SL improved swimming endurance and post-exercise locomotion, with 6′-SL generally showing the stronger effect. Metabolomic and genetic analyses linked these benefits to changes in glycogen utilization, lipid metabolism, mitochondrial activity, and AMP-activated protein kinase (AMPK)- and adenosine receptor-related signaling [137]. These findings link sialyllactose supplementation to endurance-related metabolic regulation in *C. elegans*, providing a mechanistic basis for further investigation in mammalian exercise models.

Evidence from mice has focused mainly on 6′-SL. Park et al. found that 12-week supplementation with 100 mg/kg 6′-SL increased treadmill running time, distance, total work, and grip strength [138]. These functional improvements were accompanied by greater gastrocnemius and soleus muscle mass, increased muscle-fiber diameter, and higher myosin heavy-chain expression. The findings associate 6′-SL with enhanced muscle phenotype and exercise capacity. However, because the mice did not undergo a longitudinal training program, the study does not show that 6′-SL improves training adaptation or identify the mechanism responsible for the performance effect. A related study of their group examined metabolic responses during and shortly after treadmill exercise [139]. After 12 weeks of supplementation, 6′-SL-treated mice showed lower blood lactate and higher blood glucose than controls. Their gastrocnemius muscles also contained more slow myosin heavy chain and higher levels of selected oxidative phosphorylation complexes [139]. Slow-twitch fibers have a high oxidative capacity and are relatively resistant to fatigue. These changes are therefore consistent with a shift toward a more oxidative muscle phenotype. However, both studies initially allocated 10 mice per group, whereas several functional or biochemical outcomes were analyzed in eight mice per group and some tissue or protein outcomes in only three. Random sequence generation, allocation concealment, blinding, and the selection of animals for individual analyses were not fully described, leaving uncertainty in the corresponding SYRCLE domains. These reporting limitations and small analytical samples warrant caution when interpreting the magnitude and reproducibility of the effects.

Preclinical evidence for fucosylated HMOs comes from a study of middle-aged mice. Kim et al. administered 2′-FL during a repeated forced-swimming program in 10-month-old male mice [35]. Compared with exercise alone, the combined intervention increased calf muscle mass, muscle thickness, and muscle strength. It also changed the expression of genes related to muscle protein synthesis, hypertrophy, proteolysis, and oxidative stress. These included components of the phosphatidylinositol 3-kinase (PI3K)–protein kinase B (AKT) (PI3K–AKT) pathway, transient receptor potential cation channel subfamily V member 4 (*TRPV4*), Muscle atrophy F-box protein (*Atrogin-1*), muscle-specific RING finger protein 1 (MuRF-1), and *myostatin* [35]. This study suggests that 2′-FL can modify skeletal muscle responses to repeated exercise in middle-aged mice. However, the study did not assess intestinal barrier function, microbial composition, or microbial metabolites. It therefore remains unclear whether 2′-FL acted directly on skeletal muscle or through systemic and gut-related pathways. Taken together, these preclinical studies show that selected HMOs can influence endurance behavior, muscle strength, muscle mass, fiber phenotype, and exercise-related metabolism. The current evidence therefore supports a preclinical effect on exercise capacity and muscle phenotype, but it does not yet establish a common mechanism or demonstrate accelerated post-exercise recovery.

Human evidence remains limited to two small, randomized trials and does not currently support recommending HMOs for exercise performance or recovery. Ko et al. evaluated 3 g/day 2′-FL during a 12-week intervention combining walking with energy restriction; 50 participants completed baseline testing and 41 completed follow-up [149]. Both the 2′-FL and placebo groups followed the same prescribed walking program and energy-restricted diet. The authors reported favorable changes in fat mass, preservation of fat-free mass, resting fat oxidation, and peak oxygen uptake with 2′-FL, together with a tendency toward lower IL-4 at week 6. However, group-by-time interactions were not significant for these outcomes, limiting evidence for an additional benefit over placebo. However, these findings reflect supplementation within a combined lifestyle intervention and do not isolate an effect on exercise adaptation or establish benefits in trained athletes. Estes et al. evaluated 900 mg/day 6′-SL during 12 weeks of supervised resistance training; 28 men were randomized and 19 completed the study [37]. Supplementation did not enhance gains in fat-free mass, strength, or anaerobic performance, while changes in aerobic capacity and substrate oxidation were inconsistent over time. These differences from earlier findings may reflect variation in the study population, exercise protocol, HMO structure, and dose. Benefits observed in experimental endurance models or sedentary adults undergoing weight loss may not translate to resistance-training adaptations in recreationally active men. The small sample also limits precision, although the findings may reflect a lack of additional benefit under the conditions tested. Both trials reported double-blind designs, but participant withdrawals and analyses centered on study completers raise concerns about the effects of missing data and post-randomization exclusions. Both were registered retrospectively, limiting verification that the reported analyses were prespecified. The numerous outcomes and comparisons also require caution when interpreting isolated favorable findings [37,149]. Neither trial directly assessed recovery between exercise bouts. These findings therefore provide preliminary evidence for further investigation rather than a basis for sports-supplementation recommendations.

### 5.2. Muscle Structure, Function, and Metabolic Resilience

The preservation of skeletal muscle mass, fiber organization, and force-generating capacity is fundamental to exercise performance, training adaptation, and recovery. Studies in catabolic, neuromuscular, and metabolic conditions provide complementary evidence that individual sialylated HMOs and related milk oligosaccharide preparations can influence muscle maintenance and metabolism. These findings help identify processes that may be relevant to exercise, but do not directly demonstrate recovery benefits in athletes.

Go et al. examined 6′-SL in C2C12 myotubes and mice with dexamethasone-induced muscle atrophy [36]. In cultured myotubes, 6′-SL preserved myotube diameter and suppressed markers of muscle protein degradation. In mice, supplementation maintained hindlimb muscle mass and fiber diameter and improved grip strength and rotarod performance, although treadmill endurance was not significantly altered. These findings suggest that 6′-SL can limit muscle protein degradation and preserve muscle structure under glucocorticoid-induced catabolic stress. Whether these mechanisms also contribute to muscle maintenance during exercise training requires further investigation. Clinical studies in patients with GNE myopathy, a rare inherited muscle disorder associated with impaired sialic acid synthesis and progressive muscle weakness, provide human evidence for the bioavailability and muscle-related activity of 6′-SL. Oral supplementation increased circulating free sialic acid and improved selected measures of proximal muscle strength in a randomized pilot trial [147]. A subsequent placebo-controlled trial reported more pronounced effects at 6 g/day, including improvements in selected strength outcomes and attenuation of muscle fat replacement [148]. These studies support the bioavailability and muscle-related activity of oral 6′-SL in patients with GNE myopathy. Because this disease involves impaired sialic acid synthesis, the findings cannot establish comparable effects in healthy individuals undergoing exercise training.

Complementary evidence links a bovine-milk-derived oligosaccharide mixture to lean-tissue growth and muscle metabolism. In gnotobiotic mice and piglets colonized with microbiota from undernourished infants, purified sialylated bovine milk oligosaccharides increased lean-body-mass gain and altered skeletal muscle metabolism through microbiota-dependent mechanisms [140]. The preparation was isolated from cheese whey, with 3′-SL and 6′-SL accounting for approximately 88% of its oligosaccharide content. These findings support a microbiota-dependent link between milk oligosaccharides, nutrient utilization, and tissue growth under undernutrition, providing a possible mechanism for further investigation in exercise settings. In streptozotocin-treated mice, 6′-SL also reduced circulating creatine kinase and lactate dehydrogenase and improved metabolic and inflammatory variables [141]. The study used six mice per group and reported blinded histological scoring, although blinding for the circulating biomarkers was not explicitly described. These findings suggest effects on muscle-related biomarkers under metabolic disease conditions, but do not demonstrate protection against exercise-induced muscle damage. Harris et al. linked maternal exercise to increased milk 3′-SL and showed that 3′-SL exposure during lactation contributed to improved metabolic health and cardiac function in mouse offspring [142]. This study supports a role for 3′-SL in mediating intergenerational effects of maternal exercise, rather than demonstrating improved exercise capacity or recovery in the supplemented individual.

Together, these studies indicate that 3′-SL, 6′-SL and related bovine-derived oligosaccharide mixtures can support muscle mass, fiber integrity, strength, and metabolic function across several experimental settings. The findings should be attributed to the material tested, without assuming that mixed preparations and individual HMOs are interchangeable. Their relevance to muscle repair and recovery between exercise bouts remains to be established.

### 5.3. Bone Remodeling and Skeletal Resilience

Bone density, microarchitecture, and matrix quality determine skeletal load tolerance and influence the capacity to sustain long-term physical training [146,150]. Impaired bone remodeling can increase the risk of bone stress injury, particularly during repeated high-impact loading or periods of low energy availability [150]. Evidence from growth, aging, and osteoporosis models indicates that individual HMOs, HMO blends, and bovine-milk-derived oligosaccharide preparations can influence bone formation, resorption, and strength through microbial, immune, and metabolic pathways [143,144]. These findings provide a biological basis for considering the gut–bone axis in exercise adaptation and musculoskeletal resilience.

Mechanistic support for this connection comes from gnotobiotic models of infant undernutrition, in which germ-free animals are colonized with specified microbial communities. Charbonneau et al. showed that sialylated bovine milk oligosaccharides promoted microbiota-dependent growth and altered bone morphology in mice and piglets, with increased femoral cortical thickness, cortical bone mineral density, and trabecular bone volume fraction observed in mice [140]. Cowardin et al. subsequently found that the same preparation increased femoral trabecular bone volume and cortical thickness in gnotobiotic mice [143]. These effects were attributed mainly to reduced osteoclast abundance and bone resorption and were linked to microbial succinate production, intestinal tuft-cell expansion, and type 2 immune signaling. This work identifies a microbiota–immune–bone pathway through which the tested bovine oligosaccharide preparation may preserve skeletal structure when nutrient availability and bone remodeling are disrupted. Evidence from aging and estrogen-deficiency models further supports the bone-protective potential of specific HMOs. Twelve weeks of 2′-FL supplementation attenuated bone loss and microstructural deterioration in naturally aging mice, accompanied by changes in the gut microbiota and innate immune regulation [144]. Although six mice were allocated to each group, the principal bone microarchitecture results were reported for three animals per group. The selection of these animals and the procedures for allocation concealment and blinded outcome assessment were not clearly described, leaving uncertainty in the relevant SYRCLE domains. In ovariectomized rats, 2′-FL also reduced estrogen-deficiency-associated bone loss and improved intestinal dysfunction [145]. These findings support effects of 2′-FL on bone preservation in aging and estrogen-deficiency models and provide a rationale for investigating whether similar pathways influence skeletal responses to exercise. Long-term effects on bone quality have also been observed with neutral core HMOs. Bonnet et al. examined female minipigs exposed to HMO-enriched formula during the preweaning period [146]. Neutral core HMOs improved tibial bone density, microstructure, matrix quality, and mechanical strength at one year of age, with bone strength approaching that of naturally sow-fed animals. These persistent effects indicate that early HMO exposure can influence not only bone mass but also the structural and mechanical properties that determine load-bearing capacity.

The findings also show that skeletal responses depend on HMO structure and physiological context. Sialylated bovine milk oligosaccharides reduced bone resorption in the undernutrition model, whereas neutral core HMOs produced stronger long-term effects in formula-fed minipigs [140,143]. Similarly, 2′-FL did not reproduce the effects of sialylated bovine milk oligosaccharides in one gnotobiotic model but protected bone in aging and estrogen-deficiency models [144,145]. These differences may reflect variation in HMO structure, host physiology, or microbial responses, although the available studies do not establish their relative contributions. HMO structure, developmental stage, hormonal status, and baseline microbiota should therefore be considered when interpreting skeletal outcomes. The findings provide a rationale for exercise-related bone research, but do not demonstrate improved adaptation to mechanical loading or reduced bone stress injury risk.

### 5.4. Integrated Interpretation: From Exercise Capacity to Musculoskeletal Resilience

Current evidence suggests that HMOs may support exercise in two related ways: by influencing energy use and fatigue resistance during activity, and by helping maintain muscle and bone function during repeated training. These effects may involve glycogen use, mitochondrial metabolism, protein turnover, tissue remodeling, and microbial and immune signaling. HMOs may therefore act across the gut, metabolism, muscle, and bone to support physiological resilience rather than functioning as conventional performance-enhancing nutrients. The evidence also argues against treating HMOs as a functionally uniform class. The reported effects of 2′-FL, 3′-SL, and 6′-SL differ across physiological outcomes and experimental settings, indicating that the selection of an HMO should be guided by the intended function. Sialylated structures currently show the clearest association with exercise-responsive metabolism and muscle function, whereas 2′-FL has broader links to metabolic regulation and bone preservation. Dose, habitual diet, baseline microbiota, sex, age, and training status may further determine the response. Confidence in the available findings is limited by small analytical samples, participant withdrawals, incomplete reporting of bias-control procedures, and uncertainty about prespecified analyses. These issues should be distinguished from the limited applicability of disease or developmental models to athletes. Multiple outcomes testing also increases the possibility of chance findings, while selective reporting and preferential publication of positive results remain possible but have not been established. The evidence therefore supports further investigation, with independent replication needed before consistent benefits can be inferred.

Future studies should therefore use mechanism-linked designs rather than broadly screening unrelated performance outcomes. Exercise trials should combine functional endpoints with measurements of substrate metabolism, muscle damage and restoration, gastrointestinal integrity, microbial metabolites, and immune regulation. Longer interventions should determine whether these changes improve tolerance to repeated training, preserve muscle and bone during periods of high physiological demand, or reduce interruption caused by gastrointestinal or musculoskeletal stress. Such studies would clarify whether HMOs are most effective as modulators of exercise capacity, training resilience, or recovery.

## 6. Translation into Sports Nutrition

Translation of HMOs into sports nutrition requires more than evidence of biological activity. Development decisions must connect the selected HMO structure with a defined use, an appropriate supplementation regimen, acceptable tolerability, reproducible manufacturing, and outcomes that are meaningful to athletes.

### 6.1. Selection and Supplementation Strategy

Selection of HMOs for sports nutrition should consider both exercise-related evidence and experience from adult supplementation. Among the HMOs studied directly in exercise models, 6′-SL currently has the most consistent evidence related to endurance capacity, muscle phenotype, and exercise metabolism, while 3′-SL has shown effects on endurance metabolism and exercise-responsive systemic signaling [137,138,139,142]. The evidence for 2′-FL includes exercise interventions in mice and a lifestyle intervention involving increased physical activity in adults [35,149]. LNnT has not been tested directly in exercise but has been administered alone and with 2′-FL in adult studies targeting the gut microbiota [31,118,151]. These differences support selecting the HMO according to the intended endpoint rather than treating all structures as interchangeable.

The two human exercise-related studies administered 3 g/day 2′-FL or 0.9 g/day 6′-SL for 12 weeks, whereas an adult safety trial evaluated 6 g/day 6′-SL over the same period [37,152]. In non-exercise adult studies, 2′-FL, LNnT, or their combinations were administered at 5–20 g/day for two weeks and produced dose- and participant-dependent microbiota responses [31,118,151]. A four-week trial in adults with irritable bowel syndrome further showed that a 4:1 mixture of 2′-FL and LNnT at 5 or 10 g/day altered bifidobacterial abundance and microbial metabolite profiles [118,151]. These findings identify tolerated and biologically active dose ranges but do not establish an effective dose for exercise or demonstrate that a combination is superior to an individual HMO. Exercise-specific dose-ranging studies should therefore compare individual HMOs with dose-matched combinations and identify the lowest dose that produces the intended response without increasing gastrointestinal symptoms.

Current evidence also supports repeated daily supplementation more strongly than acute use. Exercise, microbiota, and muscle-related studies have all used interventions lasting several weeks, and no controlled study has determined whether a single dose taken before, during, or after exercise produces a meaningful response [31,37,118,138,149]. Specific exercise-timing recommendations are therefore premature. Formulation evidence is available mainly from infant formula and conventional food matrices. Regulatory stability data indicate that 2′-FL and LNnT can withstand infant-formula processing and prolonged storage [153,154]. LNnT has also remained stable following pasteurization or ultra-high-temperature processing in yoghurt, ready-to-drink milk, and citrus beverages [155]. Validated analytical methods can quantify 2′-FL, LNnT, and other major HMOs in both infant formula and adult nutritional products [156]. These findings support their incorporation into processed nutrition products but do not establish stability in sports drinks, gels, or carbohydrate- and protein-containing powders. The final sports product therefore requires structure-specific evaluation of HMO recovery, processing stability, shelf life, and compatibility with other ingredients.

The practical use of HMOs in sports nutrition also requires consideration of whether other prebiotic oligosaccharides could achieve similar outcomes. GOS and FOS share several microbiota-mediated and immune-related effects with HMOs, providing a rationale for investigating their potential in sports medicine [157,158]. A human trial provided evidence for a potential sports-medicine application of GOS, showing that supplementation ′reduced bronchoconstriction during a hyperpnoea challenge used to simulate exercise-induced bronchoconstriction [159]. They are relevant alternatives for targeting microbial fermentation, but similarities in prebiotic activity do not establish equivalence in bacterial utilization, host interactions, or physiological effects. Direct comparisons under exercise conditions are therefore needed to determine whether a particular HMO offers additional benefits over GOS or FOS, taking account of dose, tolerability, and cost.

### 6.2. Production Scalability and Commercial Availability

Reliable production is necessary for both clinical research and commercial application. The construction of microbial cell factories has emerged as the dominant strategy for scalable HMO production, offering practical advantages over chemical synthesis and direct extraction from human milk in terms of cost, substrate versatility, and structural precision [40]. Engineered cell factories combine lactose uptake, nucleotide-sugar synthesis, linkage-specific glycosyltransferases, and product export to produce individual structures with controlled composition [160].

Manufacturing maturity differs among HMOs. Engineered *Escherichia coli* (*E. coli*) strains now produce 2′-FL above 140 g/L [161], LNnT and LNT above 100 g/L with successful 1000 L pilot-scale fermentation [162], demonstrating that synthetic biology-based HMO production has crossed the threshold of industrial feasibility. Sialylated HMO production has also advanced, with reported titers of 56.8 g/L for 3’-SL and 30.18 g/L for 6′-SL in 5 L fermentation systems [163,164]. These developments improve access to HMOs that are particularly relevant to exercise and muscle research, although fermentation titer alone does not establish commercial readiness. Productivity, raw-material cost, strain stability, downstream recovery, purity, and batch consistency also determine whether an ingredient can be supplied reproducibly.

Manufacturing readiness determines not only ingredient availability and cost but also the reproducibility of research and commercial products. Synthetic biology enables the production of structurally defined HMOs, but the final ingredient remains dependent on the production strain, fermentation and purification processes, salt form, purity, impurity profile, and mixture composition. The material used in a commercial product should therefore match that evaluated in supporting studies, and substantial changes in the production process may require additional analytical and safety assessment. Enzymatic and chemoenzymatic synthesis may broaden future access to rare or structurally complex HMOs, but near-term sports-nutrition development should prioritize ingredients supported by scalable production and validated characterization. These manufacturing attributes provide the foundation for regulatory evaluation, safety specifications, and product-quality control.

### 6.3. Safety, Regulatory, and Product Quality Considerations

Adult studies indicate that the HMOs tested to date are generally well tolerated at the investigated doses [31,118]. However, tolerability during strenuous exercise remains insufficiently characterized because exercise itself can provoke gastrointestinal symptoms. Sports trials should therefore evaluate HMOs under their intended conditions of use and distinguish supplement-related adverse events from symptoms caused by exercise.

Regulatory evaluation is specific to the individual HMO, manufacturing organism, production process, purity specification, dose, food category, and target population. Safety conclusions for one source of 2′-FL, for example, do not automatically apply to other 2′-FL ingredients or HMO structures. Regulatory acceptance has nevertheless expanded to several individual HMOs and defined HMO mixtures in the United States and European Union [165,166,167,168,169,170,171]. Much of this experience originated from applications in infant formula and foods for young children, for which assessments addressed chemical identity, manufacturing organisms, impurity profiles, stability, dietary exposure, and toxicological safety. These requirements provide an important quality and safety foundation for extending HMOs into adult and sports-nutrition products.

Sports applications introduce additional considerations because the dose, food matrix, accompanying nutrients, and conditions of consumption may differ from those evaluated in early-life nutrition. Products should maintain rigorous specifications for HMO identity and purity, structurally related carbohydrates, residual substrates, fermentation-derived proteins, microbial DNA, endotoxins, heavy metals, and microbiological contamination. Exercise-specific tolerability and batch consistency should also be confirmed in the final formulation. For competitive athletes, independent batch testing through an appropriate sports-certification program is needed to reduce the risk of inadvertent exposure to prohibited substances [172]. Translation into sports nutrition can therefore build on the manufacturing and safety controls established for early-life applications while adding formulation-specific, exercise-specific, and anti-doping quality requirements.

### 6.4. Priorities for Future Trials

The most immediate research priority is to determine whether HMOs can reduce gastrointestinal stress during prolonged or strenuous exercise. Initial trials should recruit endurance athletes with recurrent exercise-associated gastrointestinal symptoms and use randomized, double-blind, placebo-controlled designs. Standardized endurance challenges, including exercise in the heat, would provide relevant settings for evaluating efficacy and tolerability. Existing exercise studies have not directly connected HMO supplementation with gastrointestinal symptoms, epithelial injury, intestinal permeability, or microbial translocation. A prespecified, validated measure of gastrointestinal symptom severity should serve as the primary outcome, with a clinically meaningful difference defined before recruitment. Exercise completion and tolerance of carbohydrate and fluid intake could provide secondary functional outcomes. Measures of epithelial injury, permeability, microbial translocation, microbiota composition, and microbial metabolites should be used to investigate the proposed mechanism.

A second priority is to determine whether the effects of sialylated HMOs on energy metabolism and muscle phenotype observed in experimental models translate to physically active adults. Research on 3′-SL and 6′-SL should distinguish effects on exercise capacity, adaptation to repeated training, and recovery of muscle function, because these outcomes represent different physiological processes. Placebo-controlled trials could recruit trained adults during heavy training or consecutive-day exercise. Recovery studies could prioritize restoration of muscle force or exercise performance after 24–48 h, whereas 8–12-week training studies could assess gains in strength or endurance. Studies involving an energy deficit should monitor energy availability and standardize nutritional support. Each trial should prespecify one primary outcome, secondary measures, and a meaningful effect size to guide sample-size calculation. Longer studies may also examine whether muscle- and bone-related effects contribute to tolerance of sustained training loads.

A third direction concerns HMO structure and individual responsiveness. Direct comparisons among fucosylated, sialylated, and neutral-core HMOs are needed to identify which structures are most relevant to gastrointestinal, metabolic, or muscular outcomes. Comparisons between individual HMOs and defined mixtures should also determine whether combination provides an advantage. Baseline microbiota may help explain differences in response and could eventually guide more targeted supplementation. Together, these directions would clarify whether HMOs act primarily through gastrointestinal resilience, metabolic and muscular support, or coordinated effects across these systems.

## 7. Conclusions

HMOs have biological functions that overlap with several challenges relevant to exercise, including disturbances in the gut microbiota, intestinal barrier, immune regulation, energy metabolism, and musculoskeletal resilience. Experimental studies show that selected HMOs can influence endurance metabolism, muscle phenotype, and tissue maintenance. Human evidence, however, is limited to two small trials. One involved adults with overweight or obesity undergoing combined energy restriction and walking, limiting conclusions about exercise-specific effects. The other found no additional gains in fat-free mass, strength, or anaerobic performance during resistance training. Direct effects on exercise-induced gastrointestinal stress, training adaptation, and post-exercise recovery remain insufficiently established. The expanding availability of structurally defined HMOs through microbial fermentation, together with existing adult safety and regulatory experience, provides a practical basis for further sports-nutrition research. Translation should remain structure-specific and should determine whether individual HMOs or defined combinations produce meaningful benefits under exercise conditions. Until such evidence is available, HMOs are best regarded as candidate ingredients for supporting gastrointestinal, metabolic, and musculoskeletal resilience rather than established performance-enhancing supplements.

## Figures and Tables

**Table 1:** Exercise-related and supportive evidence for HMOs and related milk oligosaccharide preparations.

Study	Model/Population, Sex, Age, Training Status, and Sample Size	Design and Exercise Protocol	Material, Dose, Route, and Duration	Main Endpoints and Findings	Adverse Effects	Key Limitations
Arellano Spadaro et al. [137]	Adult *C. elegans*; no sex; *n* varied by assay; no training	Controlled supplementation; 120-min swimming challenge	Dietary 3′-SL or 6′-SL, 0.2–2 mg/mL; larval-to-adult exposure	Improved endurance and post-swimming locomotion; altered mitochondrial and energy metabolism	NR*	Non-vertebrate model; developmental exposure
Park et al. [138]	Male C57BL/6J mice, approximately 7 weeks at treatment initiation; 10/group, analyzed *n* = 3–8/group; no training	Randomized controlled study; treadmill exhaustion and grip testing	Oral 6′-SL, 100 mg/kg, 5 days/week for 12 weeks	Increased endurance, grip strength, muscle mass, and fiber diameter	NR*	Small analytical samples; blinding and sample selection unclear
Park et al. [139]	Male C57BL/6J mice, approximately 7 weeks at treatment initiation; 10/group, analyzed *n* = 3–8/group; no training	Randomized controlled study; 20-min progressive treadmill challenge	Oral 6′-SL, 100 mg/kg, 5 days/week for 12 weeks	Lower post-exercise lactate; higher glucose and selected oxidative muscle proteins	NR*	Biomarker outcomes; functional recovery not assessed
Kim et al. [35]	Male CD-1 mice, 10 months; *n* = 60; repeated swimming	Controlled study; daily 2-min forced swimming, 30 min after supplementation	Oral 2′-FL, 200, 400, or 800 mg/kg once daily for 28 days	Increased muscle mass, thickness, and strength; altered protein metabolism and oxidative-stress markers	NR*	Middle-aged mouse model; intestinal mechanisms untested
Go et al. [36]	Male C57BL/6N mice, approximately 6 weeks at treatment initiation; *n* = 16 (4/group); dexamethasone-induced atrophy; no training	Controlled disease model; motor-function testing	Oral 6′-SL, 100 or 500 mg/kg for 10 weeks; dexamethasone administered during the final 2 weeks	Preserved muscle mass and fiber size; improved selected motor outcomes, but not treadmill endurance	NR*	Pharmacological atrophy model; exercise recovery untested
Charbonneau et al. [140]	Male mice, 5 weeks; mouse *n* varied; neonatal piglets, *n* = 3/group in main experiment; sex not specified; no training	Controlled gnotobiotic undernutrition experiments; no exercise	Dietary S-BMO, predominantly 3′-SL/6′-SL; concentration not specified; mice 5 weeks, piglets 6 days	Increased mouse lean-mass gain and piglet growth; altered tissue metabolism	NR*	Small groups; developmental model; bovine mixture
Tran-Duc et al. [141]	Male ICR mice, approximately 14 weeks at intervention; *n* = 6/group in methods; no training	Randomized streptozotocin model; no exercise	Intraperitoneal 6′-SL, 25 or 75 mg/kg/day for 5 days	Improved metabolic and inflammatory measures; reduced circulating CK and LDH	NR*	Non-oral administration; biomarkers do not establish muscle protection
Harris et al. [142]	Female mouse dams and male/female offspring; neonatal supplementation; *n* varied by experiment; offspring sedentary	Maternal exercise, cross-fostering, and offspring supplementation	Oral 3′-SL during nursing; age-adjusted dosing, principally 600 nmol/day; follow-up into adulthood	Maternal exercise increased milk 3′-SL; supplementation improved selected offspring metabolic and cardiac outcomes	NR*	Intergenerational effects; no athlete-recovery evidence
Cowardin et al. [143]	Male gnotobiotic mice, approximately 5 weeks; *n* = 5–10/group; no training	Controlled undernutrition model; no exercise	Dietary S-BMO; dietary concentration not specified; up to 42 days	Increased trabecular volume and cortical thickness; reduced bone resorption	NR*	Mixed preparation; no mechanical-loading intervention
Li et al. [144]	Female mice, approximately 14 months; young controls 8 weeks; *n* = 6/group, bone analyses *n* = 3/group; no training	Controlled aging study; no exercise	Oral 2′-FL, 500 mg/kg/day for 12 weeks	Preserved bone density and microarchitecture; altered microbial and immune measures	NR*	Small analytical samples; blinding and sample selection unclear
Su et al. [145]	Female Sprague–Dawley rats, 12 weeks at purchase; *n* = 32 (8/group); ovariectomy model; no training	Controlled estrogen-deficiency model; no exercise	2′-FL, 480 mg/kg once daily for 11 weeks	Reduced bone loss and intestinal dysfunction	NR*	Disease model; exercise-related bone outcomes untested
Bonnet et al. [146]	Female minipigs; preweaning intervention, assessment at 1 year; *n* = 56 total; no training	Randomized feeding study; no exercise	Formula containing a sialylated HMO blend (0.68 g/L), a neutral HMO blend (4 g/L), or a combined blend (4 g/L), from postnatal day 10 to week 11	Neutral-HMO blend improved tibial density, structure, matrix quality, and strength	NR*	Early-life exposure; mixture-specific effects
Park et al. [147]	Adults with GNE myopathy; 10 men/10 women, 24–58 years (36.1 ± 7.4 years); *n* = 20, plus pharmacokinetic cohort *n* = 10; training status not reported	Randomized pilot trial; no prescribed exercise	Oral 6′-SL, 3 or 6 g/day in three divided doses for 96 weeks; placebo recipients switched to 3 or 6 g/day after week 12	Increased circulating sialic acid; selected strength and MRI findings favored the higher dose	Transient GI discomfort	Small trial; disease-specific mechanism; limited long-term placebo comparison
Park et al. [148]	Men and women with GNE myopathy; 6 men/5 women, 25–62 years (38.6 ± 12.4 years); *n* = 11; training status not reported	Randomized placebo-controlled trial; no prescribed exercise	Oral 6′-SL, 6 g/day for 48 weeks	Most strength outcomes showed no group difference; posterior-thigh MRI fat-fraction progression favored 6′-SL	No safety concerns reported	Very small sample; multiple outcomes; disease-specific population
Ko et al. [149]	Sedentary adults with overweight/obesity; 18 men/23 women among completers; 38 ± 13 years; 50 baseline, 41 completed	Double-blind RCT; 10,000 steps/day, 5 days/week, plus approximately 300 kcal/day restriction	Oral 2′-FL, 3 g/day for 12 weeks	No significant additional benefits over placebo for body composition, resting substrate oxidation, aerobic capacity, or inflammatory cytokines	No adverse side effects reported	Attrition; retrospective registration; multiple outcomes; combined lifestyle intervention
Estes et al. [37]	Recreationally active men, 24.4 ± 6.0 years; 28 randomized, 19 completed	Double-blind RCT; supervised resistance training, 3 sessions/week	Oral 6′-SL, 900 mg/day for 12 weeks	No additional gains in fat-free mass, strength, or anaerobic capacity; inconsistent aerobic/metabolic effects	Mild, infrequent symptoms; no group difference or related withdrawals	Attrition; retrospective registration; multiple outcomes; small male-only sample

NR*, no systematic adverse-effect assessment identified in the material reviewed. BMC, bone mineral content; CK, creatine kinase; GI, gastrointestinal; LDH, lactate dehydrogenase; MRI, magnetic resonance imaging; RCT, randomized controlled trial; S-BMO, sialylated bovine milk oligosaccharides; 2′-FL, 2′-fucosyllactose; 3′-SL/6′-SL, 3′-/6′-sialyllactose.

## Data Availability

No new data were created or analyzed in this study. Data sharing is not applicable to this article.

## Abbreviations

The following abbreviations are used in this manuscript:

2′-FL	2′-fucosyllactose
3’-SL	3′-sialyllactose
3-FL	3-fucosyllactose
6′-SL	6′-sialyllactose
AMPK	AMP-activated protein kinase
*B. bifidum*	*Bifidobacterium bifidum*
*B. breve*	*Bifidobacterium breve*
*B. infantis*	*Bifidobacterium longum* subsp. *infantis*
*B. pseudocatenulatum*	*Bifidobacterium pseudocatenulatum*
*C. elegans*	*Caenorhabditis elegans*
*C. jejuni*	*Campylobacter jejuni*
*E. coli*	*Escherichia coli*
*E. hallii*	*Eubacterium hallii*
*F. prausnitzii*	*Faecalibacterium prausnitzii*
Fuc	ʟ-fucose
Gal	ᴅ-galactose
Glc	ᴅ-glucose
GlcNAc	N-acetylglucosamine
HMOs	Human Milk Oligosaccharides
I-FABP	Intestinal Fatty Acid-Binding Protein
LDFT	Lacto-N-difucohexaose
LNnT	Lacto-N-neotetraose
LNT	Lacto-N-tetraose
LPS	Lipopolysaccharide
NeuAc	N-acetylneuraminic acid
NF-κB	Nuclear Factor kappa-light-chain-enhancer of activated B cells
SCFA	Short-Chain Fatty Acid
TLRs	Toll-like receptors
TNF-α	Tumor Necrosis Factor-alpha
*V. atypica*	*Veillonella atypica*
